# Preparation of Targeted Lignin–Based Hollow Nanoparticles for the Delivery of Doxorubicin

**DOI:** 10.3390/nano9020188

**Published:** 2019-02-02

**Authors:** Yu Zhou, Yanming Han, Gaiyun Li, Sheng Yang, Fuquan Xiong, Fuxiang Chu

**Affiliations:** 1 Research Institute of Wood Industry, Chinese Academy of Forestry, Xiangshan Road, Beijing 100089, China; zhouyu_sky@126.com (Y.Z.); ligy@caf.ac.cn (G.L.); yangsheng@criwi.org.cn (S.Y.); 2 College of Materials Science and Engineering, Central South University of Forestry and Technology, Changsha 410004, China; xiongfuquan@126.com

**Keywords:** lignin, nanoparticles, targeted materials, drug delivery

## Abstract

Due to their exceptional absorption capacity, biodegradability, and non-toxicity, nanoparticles (NPs) from lignin have emerged as vehicles for inorganic particles and drug molecules. However, the method for preparing targeted lignin particles is still complex and lacks sufficient research. Herein, a succinct strategy was proposed for the preparation of targeted lignin-based drug delivery NPs to load Doxorubicin Hydrochloride (DOX). The lignin hollow NPs (LHNPs) were used as a platform for the preparation of targeted delivery material by incorporating magnetic NPs and folic acid (FA) via layer-by-layer self-assembling. The results showed that the surface of LHNPs was covered uniformly by Fe_3_O_4_ NPs and grafted with folic acid. The folic-magnetic-functionalized lignin hollow NPs (FA-MLHNPs) could respond to magnetic field and folic acid receptors. In addition, the targeting performance of the FA-MLHNPs increased the cellular uptake of NPs in the case of HeLa cells. This research not only supported the modified NPs platform as a highly efficient nano-delivery method but also provided a facile approach to utilize renewable lignin biomaterials.

## 1. Introduction

Antineoplastic antibiotic drugs are highly potent against cancer, yet also toxic, and the mechanisms of action can result in morbidity [1,2]. Small molecule drugs can simply diffuse and distribute freely throughout the body [3], thereby resulting in undesirable side effects that can limit the achievement of proper doses in humans [4]. It was, therefore, necessary to apply a drug delivery system to obstruct drug and normal tissues, and then directly target the cancerous tissues. Novel nanomaterials and nanocarriers, types of silicon-based nanoparticles [5], polymeric nanoparticles [6], polymersomes [7] and liposomes [8], have been used as drug delivery systems to protect the drug from rapid degradation after systemic distribution and target cancer cells using ligands. Consequently, the drug reaches the tumor site at therapeutic concentrations and possible side effects can be reduced [9].

Lignin is a phenolic polymer derived from phenylpropanoid units, specifically syringyl, guaiacyl and *p*-hydroxyphenolmonomers [10,11], with many special properties, such as resistance to decay and biological attack, UV protection, high stiffness, antibacterial and low toxicity. Thereby, lignin was used in the field of drug carriers after being nano-treated [12,13]. Li et al. prepared a novel lignin-based microsphere by self-assembly, with sodium lignosulfonate and cetyltrimethyl ammonium bromide aggregated into uniform colloidal spheres, and the microsphere was used as shell material to encapsulate avermectin (AVM) [14]. The nanoparticle exhibited controlled release and anti-photolysis performance for AVM. Chen et al. fabricated lignin-based pH-responsive nano-capsules, which could be readily loaded with hydrophobic coumarin-6 via an interfacial miniemulsion polymerization. Moreover, the release of encapsulated coumarin-6 could be controlled by varying pH [15]. Dai et al. investigated alkali lignin, bioactive molecule resveratrol (RSV), and Fe_3_O_4_ magnetic nanoparticles to form magnetic RSV-loaded lignin nanoparticles by self-assembly [16]. Figueiredo et al. developed a magnetic lignin NPs that were able to efficiently load poorly water-soluble drugs and could improve their release profiles at pH 5.5 and 7.4 in a sustained manner [17]. However, a succinct strategy for preparing functional nanoparticles using raw lignin was needed.

In previous work, our group has developed one hollow nanoparticles hole via self-assembly using raw enzymatic hydrolysis lignin (greater chemical activity and good solubility in ubiquitous organic solvent) [18]. The results showed that the nanoparticles exhibited low density, large surface area, high surface permeability, well-proportioned and stable microstructure. Therefore, it has the potential to produce a high value drug delivery systems [19]. Previous research has found that the penetration of NPs into a tumor interstitium was impeded by an abnormal transcapillary pressure gradient and interstitial fluid pressure induced by angiogenesis [20]. This study provide a succinct strategy for synthesizing a lignin-based green targeted drug delivery nanoparticles platform aimed at loading the hydrophilic drug doxorubicin hydrochloride (DOX). Herein, the modification of targeting molecules on the surface of nanoparticles may address these obstacles by providing a guide to achieve a controllable biodistribution and targeted in vitro drug release.

## 2. Materials and Methods

### 2.1. Materials

Enzymatic hydrolysis lignin (EHL) was acquired from Hong Kong Laihe Biotechnology Co., Ltd. (Hong Kong, China) The hydroxyl content, the number-average molecular mass, and polydispersity of EHL are 2.67 mmol/g, 1430 g/mol, and 1.22, respectively. Doxorubicin hydrochloride was purchased from Shanghai Macklin Biochemical Co., Ltd. (Shanghai, China) Purity is higher than 98%. Tetrahydrofuran (THF) of analytical-grade purity was provided by Beijing Chemical Reagent Company (Beijing, China). Folic acid was supplied by Chembase Co., Ltd. (Shanghai, China) Iron (II) chloride tetrahydrate and Iron (III) chloride and albumin from bovine serum were purchased from Shanghai Macklin Biochemical Co., Ltd. (Shanghai, China) Poly (dimethyl diallyl ammonium chloride) solution (PDAC, Mw < 100,000, 35 wt.% in water, 100–200 CP (25 °C)) was provided by Shanghai Aladdin Bio-Chem Technology Co., Ltd. (Shanghai, China).

### 2.2. Synthesis of Lignin Hollow NPs

The preparation of lignin hollow nanoparticles (LHNPs) was achieved according to the typical self-assembly method published by Xiong et al. [18]. Briefly, 10 mg EHL was dissolved in THF to prepare a 1 mg/mL EHL–THF solution at room temperature, and the mixture solution was stirred at 800 rpm under magnetic stirring. Thereafter, 40 mL of water was then gradually dropped into the mixture solution at a rate of 4 mL/min, then lignin molecules were self-assembled into colloidal spheres.

### 2.3. Preparation of Magnetic NPs (MLHNPs)

The synthesis of Fe_3_O_4_ NPs was performed according to a previous method [21]. Firstly, 100 mL of deionized water and certain amounts of FeCl_3_·6H_2_O and FeCl_2_·4H_2_O (n(Fe^3+^): n(Fe^2+^) = 1:2) were added into a 250 mL four-necked flask fitted with a mechanical overhead stirrer, and inlet of nitrogen gas. Subsequently, ammonium hydroxide (1M) was injected into the four-necked flask until the pH was 9, and then the mixture was stirred for 15 min at 40 °C. Finally, the Fe_3_O_4_ NPs was dispersed to prepare a solution after that was separated and washed several times and set aside for 5 min. Then the solution was maintained for further use, and the concentration of Fe_3_O_4_ NPs was 2.5 × 10^−3^ mg/mL.

### 2.4. Synthesis of LHNPs Functionalized with Magnetic NPs and Folic Acid Molecule

The functionalized lignin nanoparticle was carried out in the following several steps: the LHNPs were first prepared, then Fe_3_O_4_ NPs solution (1 mL, 2.5 × 10^−3^ mg/mL) was added into the colloidal liquid and the mixture stirred for at least 1 h. In the next step, the surface charge of the particles was reversed by adding 2 μL of PDAC. After 30 min, using ammonia to adjust the solution to be weakly alkaline (pH < 8), folic acid (1 mL, 1 mg/mL) was added into the colloidal liquid solution. Finally, albumin from bovine serum (BSA, 10 mL, 1 mg/mL) was added to reverse the surface charge of the particles at negative ζ-potential. The obtained colloidal liquid was introduced into a dialysis bag (MWCO: 12,000-14,000, Spectrumlabs, Los Angeles, CA, USA) and then immersed in excess of (periodically replaced) deionized water to remove the free molecules and THF.

### 2.5. Drug Loading

The DOX-loaded LHNPs (LHNPs @DOX) were prepared using a co-assemble method. In brief, 1 mg of DOX was dispersed in 10 mL THF containing EHL (drug: lignin of 1:10) and stirred for 5 min. Thereafter, 40 mL of water was drop-wise into the solution at a rate of 4 mL/min, which induced the co-assembly of the lignin and DOX molecules into colloidal spheres (6 < pH < 7). After 24 h, the colloidal solution was dialyzed against deionized water to remove THF and free compound. The solution was centrifuged at 11,000 rpm for 15 min, and the absorbance of the supernatant was measured at 480 nm with a UV–Vis spectrophotometer to determine the drug concentration. Effects of different *m*_DOX_/*m*_EHL_ on drug loading and Encapsulation were showed in Table S1.

The DOX loading and encapsulation efficiency were calculated according to the following equations:

(1) $$\mathrm{Encapsulation}\;\mathrm{efficiency}(\%)=(\frac{\mathrm{DOX}\;\mathrm{loading}}{\mathrm{intial}\;\mathrm{amount}\;\mathrm{of}\;\mathrm{DOX}})\times 100$$

(2) $$\mathrm{DOX}\;\mathrm{loading}(\%)=(\frac{\mathrm{weight}\;\mathrm{of}\;\mathrm{DOX}\;\mathrm{loaded}}{\mathrm{weight}\;\mathrm{of}\;\mathrm{NPs}@\mathrm{DOX}})\times 100$$

### 2.6. In Vitro Release Studies

The in vitro release of DOX from the LHNPs was investigated using dialysis against PBS (pH 7.4 and 5.5). In brief, 1 mg of pure drug and 10 mg of drug-loaded LHNPs were immersed in 50 mL PBS and then centrifuged at a speed of 100 rpm and a temperature of 37 °C. At scheduled intervals, 5 mL of medium was collected and replaced with the same amount of fresh release medium. The samples were centrifuged at 11,000 rpm for 5 min, and the absorbance (i.e., amount of DOX released) was measured at 480 nm with a UV spectrophotometer. The average calculated values were obtained from at least three replicates.

### 2.7. Cellular Uptake of Modified LHNPs Assays

Confocal laser scanning microscopy (CLSM) was used to study the cellular uptake of three kinds of LHNPs. HeLa cells were plated and cultured in six-well plates. Thereafter, the LHNPs were FITC-labeled to track the distribution of nanoparticles during the assay. After incubation for 24 h, different concentrations (LHNPs served as a standard) of the modified LHNPs were added into the wells. After incubation for 4 and 24 h, the cells were washed three times with PBS and fixed with paraformaldehyde in PBS. Images of the uptake were obtained by CLSM.

### 2.8. In Vitro Cytotoxicity Studies

The cytotoxicity of the LHNPs was evaluated using an MTT assay. HeLa cells were cultured in a 96-well flat-bottom plate with 6 × 10^3^ cells per well. After incubation for 24 h, different concentrations of DOX, NPs or NPs@DOX were added to each well, and a powerful magnetic field was established at the periphery of each well. After incubation for 48 h, the cells were washed twice with PBS and incubated for an additional 2 d at 37 °C in fresh media. Thereafter, the media were removed, and the cells were dissolved in DMSO. The absorbance of each well was measured at 570 nm with a microplate reader.

## 3. Results and Discussion

### 3.1. The Preparation of Folic-Magnetic-Functionalized Lignin Hollow Nanoparticles (FA-MLHNPs) and Formation Mechanism of NPs

**Figure 1:**
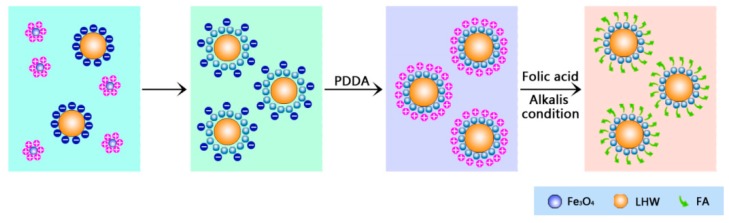
Schematic illustration of layer-by-layer self-assembly of folic-magnetic-functionalized lignin hollow nanoparticles (FA-MLHNPs).

Using the LHNPs as precursors to electrostatic adsorb Fe_3_O_4_ NPs and folic acid at different stages, lignin–based hollow targeted nanoparticles were prepared via a layer-by-layer self-assembling. Figure 1 illustrates the major experimental process in this study. Average size and ζ-potential of nanoparticles suspension was obtained by Zetasizer Nano ZS. According to the data of ζ-potential in Table 1, the ζ-potential of LHNPs and Fe_3_O_4_ NPs were −38.2 ± 9 mV and 34.7 ± 17 mV, respectively. Firstly, LHNPs and Fe_3_O_4_ NPs formed electrostatic adsorption in reaction systems to yield magnetic lignin hollow nanoparticles (MLHNPs, ζ-potential of −27 ± 7 mV). Because of the interaction with electron rich oxygen atoms in the hydroxyl groups of lignin and Van der Waals forces, approximately 5–10 nm Fe_3_O_4_ NPs (Figure S1) could be embeded inside the LHNPs via electrostatic interactions and Van der Waals adsorption [22]. Secondly, the surface charging of the particles was reversed by the adsorption of PDAC [23], and the test revealed the PDAC-coated NPs had a negative ζ-potential of 34.5 ± 5.5 mV. Deprotonation of folic acid interacted with the cationic polyelectrolyte for the carboxyl group on the molecular structure deionized to carboxy ion in an alkaline aqueous solution (Figure S2) [24]. Molecular folic acid was modified to the surface of particles by electrostatic interaction. Finally, the surface charge of the function nanoparticles were reversed by Bovine serum albumin (−20.2 ± 5). Herein, a targeted nanoparticle (FA-MLHNPs) was obtained by adding a small amount of aqueous folic acid in a weak alkaline medium.

### 3.2. FA-MLHNPs Characterization

Particle construction, particle size and size distribution were significant parameters and had relevance with the drug loading, controllable release, stability and biodistribution [25]. The quantitative data of prepared NPs are listed in Table 1. The average particle size of LHNPs, MLHNPs, and FA-MLHNPs was 286 ± 16, 302 ± 8 and 324 ± 12 nm, respectively. And the PDI values were all less than 0.25, showing moderately uniform dispersity of the prepared LHNPs.

**Figure 2:**
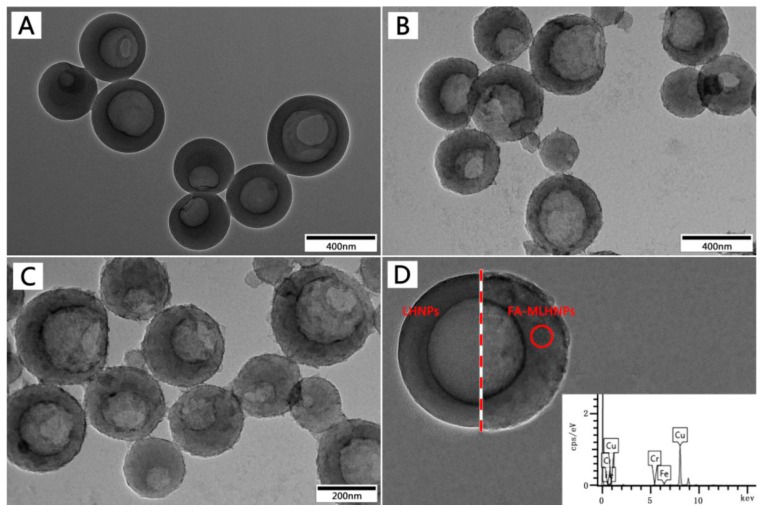
Transmission electron microscope (TEM) images of (**A**) LHNPs, (**B**) MLHNPs, (**C**) FA-MLHNPs. (**D**) Schematic comparison of the two types of LHNPs in this study, the inset is the energy spectrum of the surface of FA-MLHNPs.

The size and morphology of the LHNPs were investigated by transmission electron microscope (TEM). As shown in Figure 2, the LHNPs exhibited discrete, uniform spheres with a single hole on the surface. In addition, the images displayed a distinct contrast between the center and the shell that indicates the presence of a cavity. Compared with Figure 2A–C, showed Fe_3_O_4_ NPs uniformly and strongly embedded inside the LHNPs [22]. Figure 2D showed that the embedding of Fe_3_O_4_ NPs results in a coarser surface and no significant change was found after the surface was grafted with folic acid. In addition, the grafting yield of folic acid was counted (27.5 ± 6%) by means of ultraviolet spectroscopy. Though treatment, the modification method proposed in this study enabled the surface of LHNPs covered more uniformly by Fe_3_O_4_ NPs, and the folic acid was grafted onto the NPs surface through a more straightforward approach.

**Figure 3:**
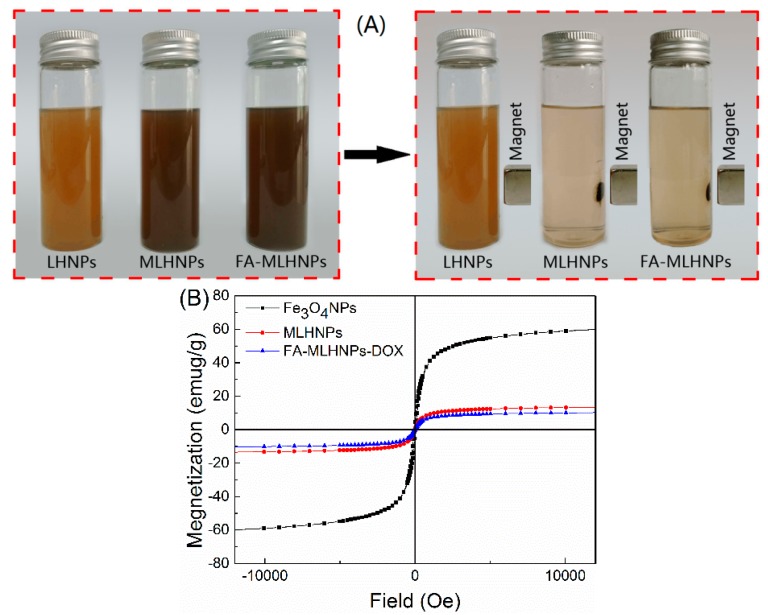
(**A**) The appearances of the lignin nanoparticles and the separation of the Magnetic particles with a magnet. (**B**) Magnetization curves of Fe_3_O_4_ NPs, MLHNPs and FA-MLHNPs.

The magnetic properties of FA-MLHNPs were also studied. The iron content of the FA-MLHNPs was previously obtained by atomic adsorption spectrometry. The result showed that FA-MLHNPs were embedded with Fe_3_O_4_ NPs, resulting in an iron content of 6.59 wt.% of the solids. In Figure 3A, the solutions became clear after introducing a magnetic field, suggesting that the Fe_3_O_4_ NPs were immobilized in the magnetic LHNPs. The magnetic hysteresis loops of NPs were comparatively measured with a Quantum Design MPMS-XL-7 superconducting quantum interference device at room temperature, which were provided by the magnetization versus magnetic field curves in Figure 3B. The saturation magnetization intensities of FA-MLHNPs, MLHNPs and Fe_3_O_4_ NPs were about 10.25, 13.5 and 60.15 emu/g, respectively. It was obvious that the saturation magnetization intensities of Fe_3_O_4_ NPs were higher than the others. The magnetization loss of the FA-MLHNPs and MLHNPs were probably due to the presence of nonmagnetic organic components and the large size of the Fe_3_O_4_ loaded LHNPs [22,26]. Although the saturation magnetization decreased, the results showed that the superparamagnetic properties were preserved after the encapsulation of Fe_3_O_4_ NPs into LHNPs.

**Figure 4:**
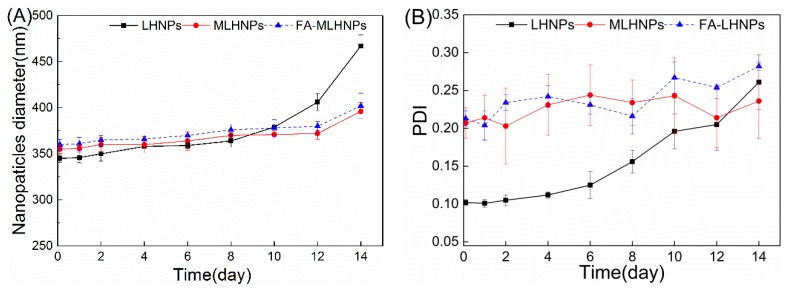
Stability of LHNPs after 14 day incubation in PBS (pH 7.4) at 37 °C, effects on the average diameter (**A**), PDI (**B**).

The biological stability of NPs was needed to be examined. In Figure 4, the stability of the LHNPs was evaluated at a physiological pH by incubating the LHNPs with PBS (pH 7.4) at 37 °C for 14 days. The LHNPs maintained a constant size for 10 days in PBS (Figure 4A), suggesting a high colloidal stability without aggregate formation. And small variations of the PDI values were observed for MLHNPs and FA-LHNPs over time (Figure 4A). However, the long-term immersion of the nanoparticles in PBS affected the double-layer structure, thereby causing particle aggregation. Thus, we observed an increase in the average diameter and the PDI after 10 days.

Therefore, the magnetic LHNPs could respond to the magnetic fields, which is an important feature that could be used for cancer therapy and diagnosis, magnetic targeting, and magnetic resonance imaging [27,28]. The Fourier transform infrared (FTIR) spectrum (S2) showed the absorption band at around 574 cm^−1^ was attributed to the stretching vibration of Fe-O in Fe_3_O_4_ NPs [29]. The peak at 1640 cm^−1^ and 1620 was the characteristic bands of folic acid [30]. Therefore, the FA-MLHNPs can be used as a promising vehicle for delivery of drug molecules to a specific target in the body by active targeting (folic acid) and passive targeting (magnetic NPs).

### 3.3. NPs Cellular Uptake

**Figure 5:**
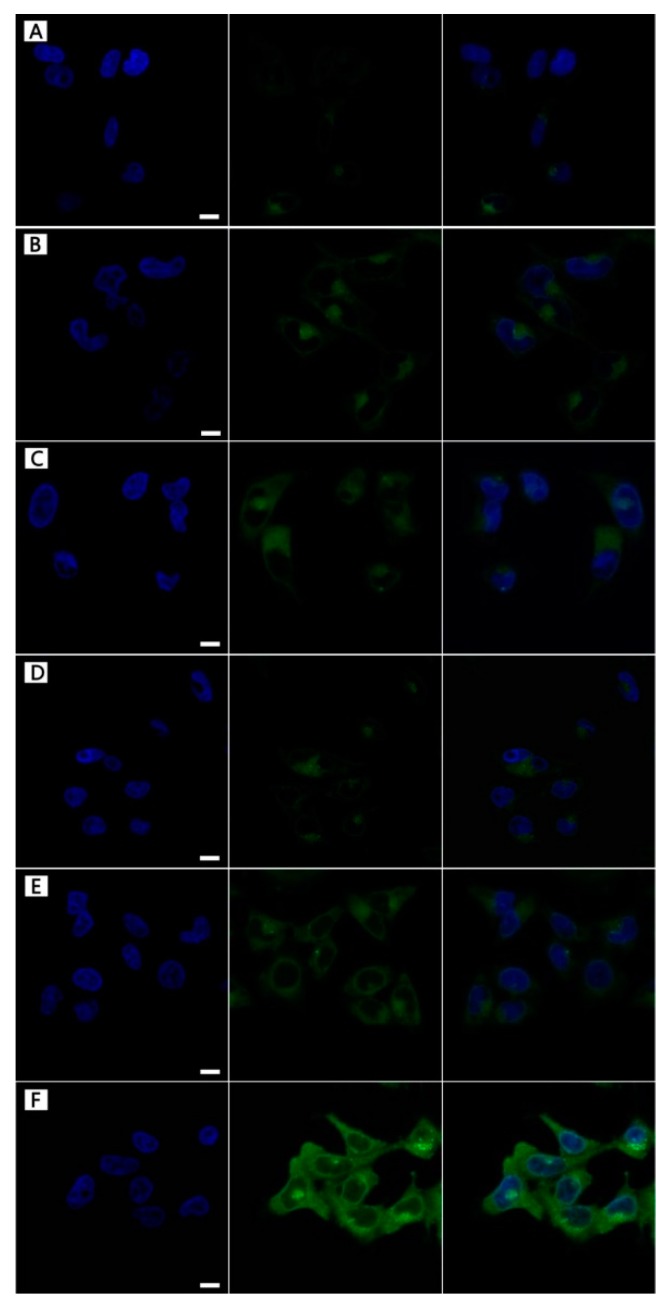
The localization of FITC-modified LHNPs from CLSM: FITC–LHNPs (**A**–**D**), FITC–MLHNPs (**B**,**E**), FITC–FA–MLHNPs (**C**–**F**) in HeLa cells for 4 h (**A**–**C**) and 12 h (**D**–**F**). Bar: 1 μm.

More descriptions of Figure 5 were added in the paragraph. In order to intuitively observe the targeting behavior of FA-MLHNPs for HeLa cell, the three LHNPs were labeled with FITC, obtained FITC–LHNPs, FITC–MLHNPs, and FITC–FA–MLHNPs. In Figure 5, to avoid the interfering of the fluorescence from FITC-labeled NPs attaching on the surface of cell membrane, the images of the HeLa cell nucleus were selected for a 3-dimensional reconstruction of the Z-axis. After the three modified LHNPs incubation periods with HeLa cells for 4 h and 24 h in a magnetic field, the green fluorescence of the FITC-FA-MLHNPs group was the strongest, and MLHNPs were higher compared with the LHNPs group. Compare A and D, B and E, C and D, the fluorescence at 24 h was significantly better than at 4 h for all kind LHNPs. These results are due to the more rapid uptake of MLHNPs than LHNPs with an external magnetic field after adsorbing Fe_3_O_4_NPs. In addition, as the excess expression of FR on HeLa cells, the FA on the FA-MLHNPs could specifically bind to FR on HeLa cell, which could enhance the capacity of cellular uptake against HeLa cells [30]. Thus, the LHNPs, swiftly engulfed by HeLa cells, appeared heavily targeting after being modified with Fe_3_O_4_ NPs and folic acid. Additional, incubation time prolonging was beneficial for the uptake to compare the fluorescence of the two time periods.

### 3.4. In Vitro Drug Loading

**Figure 6:**
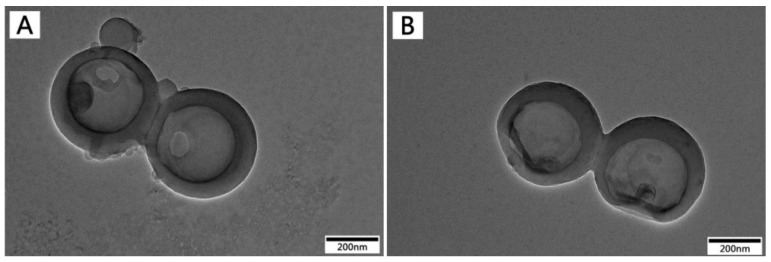
TEM images of (**A**) LHNPs@DOX, (**B**) FA-MLHNPs@DOX.

The water-soluble drug Doxorubicin hydrochloride was used as a model to study the potential application of LHNPs as a vehicle for drug delivery. DOX has a strong side-effect because of its non-specific distribution. It can widely accumulate in normal tissue, such as the heart, liver and kidney [31]. Therefore, it was necessary to develop the carriers of DOX. The TEM images of Figure 6 showed that DOX could be successfully loaded into the cavity of the hollow nanoparticles and crystallized after drying (The TEM imaged X-ray diffraction (XRD) pattern of the DOX crystals is presented in Figure S3). The sizes and PDI of DOX-loaded NPs prepared with different DOX/EHL ratios are shown in Table S1. The sizes of drug-loaded LHNPs were closer to that of blank LHNPs (286 ± 8 nm). The PDI values (<0.3) suggested a moderately uniform dispersity of the drug-loading LHNPs. The optimal DOX/EHL ratio was 1:10 with a drug loading of 8.79 ± 0.71% and an encapsulation efficiency of 67.5 ± 6%. In addition, it was found that the encapsulation efficiency of DOX-loading, which was prepared through adding the drug and lignin in THF and then added drops of water, was 67.5 ± 6%. However, the encapsulation efficiency by adding DOX to colloidal LHNPs solution to form drug-loaded LHNPs was 49.4 ± 7%. This likely caused a strong interaction between the carrier material and the drug molecules in the preparation of DOX-loading NPs [32]. In addition to adsorption action with DOX, the hydrogen bonding, and the π−π stacking also enhanced the interaction between DOX and EHL, because the lignin molecules have a similar polyphenolic structure to DOX. In addition, the ζ-potential values were −38.2 ± 9 mv and 22.7 ± 9 for LHNPs and water-DOX solution, respectively. Herein, the water-soluble drug Doxorubicin hydrochloride could successfully load into the lignin hollow nanoparticles by electrostatic attraction, hydrogen bond, and π–π interactions.

### 3.5. In Vitro Release Studies

**Figure 7:**
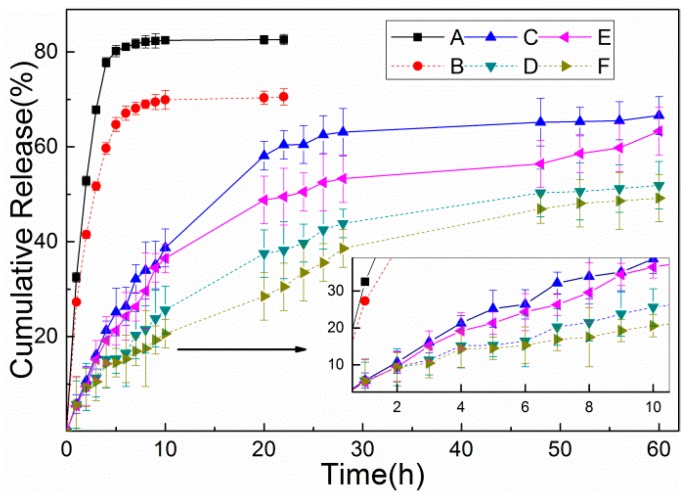
Free Doxorubicin Hydrochloride (DOX) solution was assayed as a control in (**A**) pH 5.3 and (**B**) pH 7.4 PBS. Accumulative releases of DOX from LHNPs@DOX in (**E**) pH 5.3 and (**F**) pH 7.4 PBS. And In vitro release of DOX from FA-MLHNPs@DOX in (**E**) pH 5.5 and (**F**) pH 7.4 PBS.

The drug release profiles of DOX from the three NPs at 37 °C were evaluated in two different aqueous buffer solutions in order to simulate the tumor microenvironment or intracellular endosomes (pH 5.5) and the physiological pH (pH 7.4). For the same NPs, DOX-loading NPs released DOX slightly rapidly in acidic condition, compared with the neutral condition. As shown in Figure 7, 77.8% and 59.7% of free-DOX were released within 4 h in pH 5.5 and 7.4 buffer solution, respectively. In contrast, the presence of the lignin shell prevented the release of DOX from LHNPs, and only 21.3% and 15.2% of DOX was leaked into the buffer solution over 4 h. However, due to the existence of magnetite NPs and folic acid, the DOX release decreased to 19% and 13.4% at the same time interval. In less than 30 h, the DOX from two drug-loading NPs was released smoothly under different pH conditions. This is primarily because the first release of the DOX adsorbed on the surface of the NPs and then from the cavity as the NPs form a loose sphere due to soaking in the polar medium [33]. The release curve showed the release of DOX from FA-MLHNPs@DOX was slower than that of LHNPs@DOX as the surface modification reduced the contact area between the nanoparticles aqueous buffer solutions, which delayed the release of the NPs. Furthermore, DOX was a molecule with functional groups (amine and hydroxyl groups) to interact with the magnetite surface, which limited to the release of DOX [34,35]. In summary, while both of the two kinds of nanoparticles showed a great degree of control in the release of DOX, FA-MLHNPs were better than LHNPs as the surface modification, which gave a synergistic efficacy in drug release.

### 3.6. In Vitro Cytotoxicity Studies

**Figure 8:**
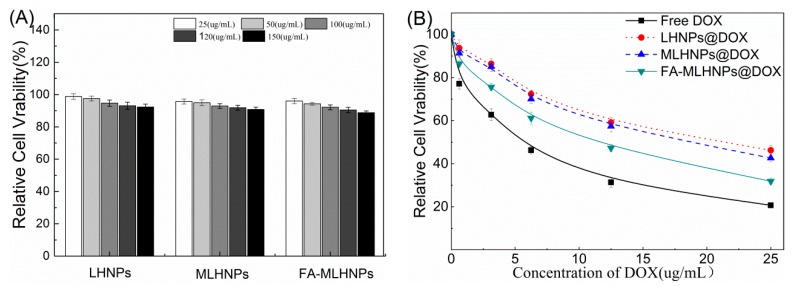
Viability of the HeLa cells after incubated with (**A**) LHNPs, MLHNPs and FA-MLHNPs and (**B**) LHNPs@DOX, LHNPs@DOX and FA-MLHNPs@DOX for 48 h at 37 °C.

Although the NPs were made of the same starting material, the surface modification altered their compositions and surface, aggregation behavior, charge and size, resulting in potential changes in cell-nanoparticle interactions and cytotoxicity in different cell lines [36,37]. Thus, the cytotoxicity of different LHNPs at different concentrations was evaluated after incubation of 48 h by MTT assay using Hela cancer cell lines. As shown in Figure 8A, all the tested concentrations of the three types of LHNPs showed very low toxicity to HeLa, and the survival rate remained above 88%. However, the higher concentration of the nanoparticles showed an increased toxicity to the cells. By contrast, the higher toxicity of LHNPs modified with Fe_3_O_4_ NPs could be correlated with highly reactive hydroxyl or superoxide radicals [38,39]. Figure 8B shows that free DOX exhibited serious side effects on the HeLa cells because DOX could passively diffuse through the cell membrane easily into the cytoplasm and quickly accumulate in the nucleus. Combined with the result of the NPs uptake, this tendency of toxicity illustrated that the internalization of the modified LHNPs@DOX was more effective in FR over-expressing HeLa cells in a magnetic field. In general, the results showed that the three LHNPs caused no significant cytotoxicity at the concentrations less than 150 μg/mL, and targeted FA-MLHNPs@DOX could easily enter into the cytoplasm to allow drug accumulate in the nucleus.

### 3.7. DOX-Loading NPs Cellular Uptake

**Figure 9:**
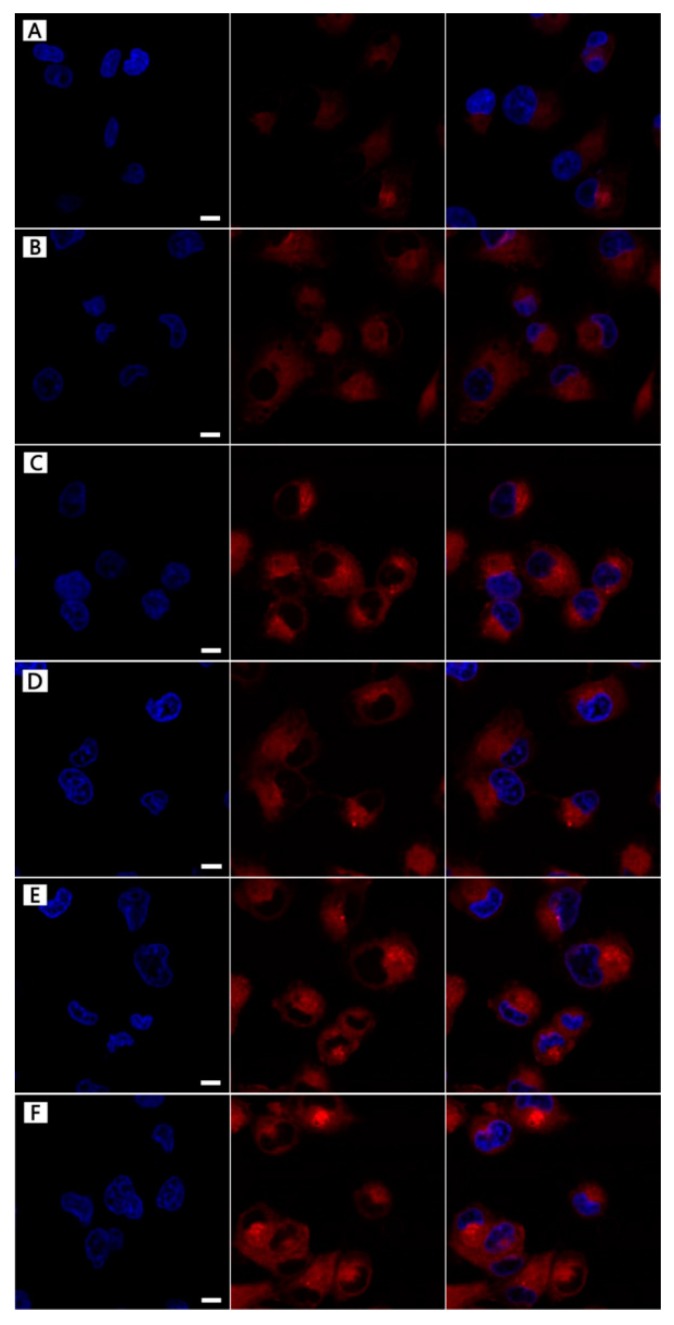
The localization of three kinds DOX loading LHNPs from CLSM: LHNPs@DOX (**A**–**D**), MLHNPs@DOX (**B**–**E**), FA-MLHNPs@DOX (**C**–**F**) in HeLa cells for 4 h (**A**–**C**) and 12 h (**D**–**F**). Bar: 1 μm.

The cellular uptake of the DOX-loading NPs was examined by CLSM to test the uptaking effect by comparing the fluorescence intensity in HeLa cells at 4 h and 24 h. Figure 9 showed the CLSM images of the treated cells and the *Z*-axis canning from top was took to bottom slices. After 4 h of treatment, the three kinds of DOX-loading NPs could be successfully uptaken by the HeLa cells, with greater internalization efficiency for drug accumulating in the cytosol. However, based on the red fluorescence intensity, FA-MLHNPs@DOX was the best, the second was MLHNPs@DOX, and LHNPs@DOX was the worst. Moreover, some red punctuate fluorescence within the HeLa nucleus demonstrated the distribution of DOX, which was cultured with FA-MLHNPs@DOX. The results revealed the effect of the magnetic field and the interaction between the FR of cell membrane and the FA on the NPs, which could enhance the capacity of cellular uptake against the HeLa cells. The test results were consistent with the cellular uptake of the unloaded NPs.

## 4. Conclusions

A lignin-based double targeted drug delivery NPs (FA–MLHNPs) platform was successfully synthesized via layer-by-layer self-assembling. The FA–MLHNPs had particle sizes of approximately 300 nm, and showed low cytotoxicity. In addition, the modified NPs, coated with uniform dense magnetic NPs and a folic acid layer could enhance the capacity of cellular uptake against HeLa cells. Herein, the LHNPs could be readily loaded with DOX during a co-assembly process with 67.5 ± 6% encapsulation efficiency based on *π*–*π* interactions and electrostatic attraction. Moreover, the DOX loaded FA-MLHNPs showed a better sustained release effect and higher anticancer efficacy than the other NPs. This work demonstrated that FA-MLHNPs provides potential in targeting the delivery of antitumor drugs and applications in cancer therapy.

## Figures and Tables

**Table 1:** Average size, polydispersity (PDI) and ζ-potential of four types NPs.

Sample	ζ-Potential (mV)	Average Size (nm)	PDI
Fe_3_O_4_ NPs	34.7 ± 17	10 ± 4	0.186
LHNPs	−38.2 ± 9	286 ± 8	0.208
MLHNPs	−27.1 ± 7	302 ± 14	0.243
FA-MLHNPs	−20.2 ± 5	314 ± 11	0.294

## Supplementary Materials

The following are available online at http://www.mdpi.com/2079-4991/9/2/188/s1, Table S1 Effect of different technique on average size, PDI, drug loading and Encapsulation efficiency of LNPs. Figure S1. TEM images of Fe_3_O_4_ NPs. Figure S2. The preparation process of folic acid molecule graft for MLHNPs. Figure S3. FTIR spectra of LHNPs, MLHNPs and FA-MLHNPs. Figure S4. TEM images and XRD pattern of crystallization DOX after drying.
