# Supplementary material for: Preparation of Targeted Lignin–Based Hollow Nanoparticles for the Delivery of Doxorubicin
Source: Nanomaterials (Basel). 2019 Feb 2;9(2):188. doi: 10.3390/nano9020188 (PMC6409628; doi:10.3390/nano9020188)
Supplement: Supplementary file 1 [file nanomaterials-09-00188-s001.pdf]

Supplementary information

Preparation of Targeted Lignin–based Hollow Nanoparticles for the Delivery of Doxorubicin

Yu Zhou[1], Yanming Han[1*], Gaiyun Li[1], Sheng Yang[1], Fuquan Xiong[2], Fuxiang Chu[1*]

[1] Research Institute of Wood Industry, Chinese Academy of Forestry, Xiangshan Road, Beijing, 100089, China

[2] College of Materials Science and Engineering, Central South University of Forestry and Technology, Changsha, 410004, China

[*]Corresponding author: Yanming Han, Fuxiang Chu

Address: NO.1 Dongxiaofu Xiangshan Road, Haidian District, 100091, Beijing, China.

Email: hanym@caf.ac.cn (Y. M. Han), chufuxiang@caf.ac.cn (F. X. Chu).

Tel.: +86-10-62889433; Fax: +86-10-62889433;

The number of pages: 6

The number of figures: 4

The number of and tables: 1

Table S1 Effect of different m_{DOX}/m_{EHL} on average size, PDI, drug loading and Encapsulation efficiency of LNPs

DOX /EHL	AverageSize(nm)	PDI	Drug loading (%)	Encapsulation efficiency (%)
1/50	285 ± 6	0.213	1.93 ±0.22	68.9±8
1/20	284 ± 7	0.213	4.50±0.65	66.1±10
1/15	289 ± 8	0.213	6.22±0.42	69.6±5
1/10	290 ± 5	0.213	8.79±0.71	67.5±6
1/7	286 ± 7	0.213	10.53±1.83	57.7±11

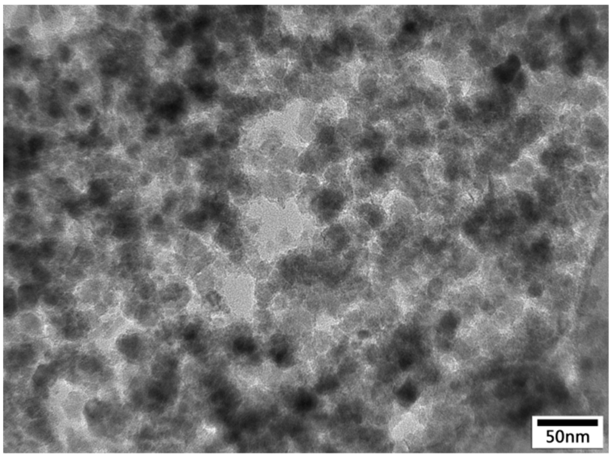

Figure S1. TEM images of Fe_3O_4 NPs

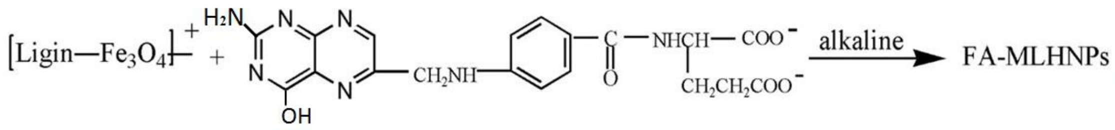

Figure S2. The preparation process of folate molecule graft for MLHNPs

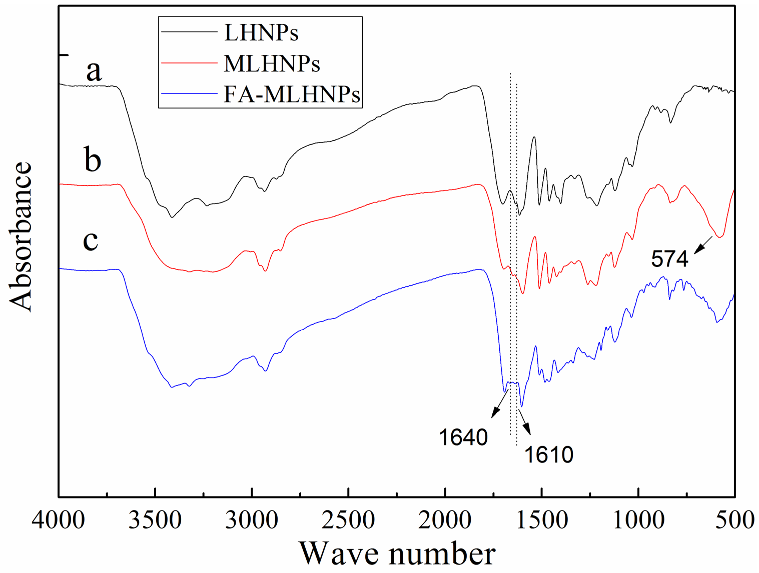

Figure S3. FTIR spectra of LHNPs, MLHNPs and FA-MLHNPs

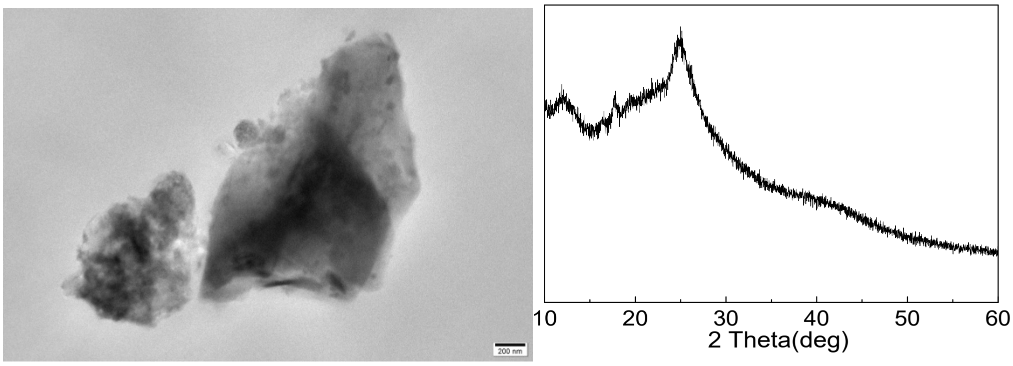

Figure S4.TEM images and XRD pattern of crystallization DOX after drying
